# Risk factors of cartilage lesion after anterior cruciate ligament reconstruction

**DOI:** 10.3389/fcell.2022.935795

**Published:** 2022-09-08

**Authors:** Zirong Huang, Jiaming Cui, Mingjin Zhong, Zhenhan Deng, Kang Chen, Weimin Zhu

**Affiliations:** ^1^ Department of Sports Medicine, Shenzhen Second People’s Hospital, The First Affiliated Hospital of Shenzhen University, Shenzhen, Guangdong, China; ^2^ Clinical Medical College, Guangzhou Medical University, Guangzhou, China; ^3^ Guangdong Key Laboratory of Tissue Engineering, Shenzhen Second People’s Hospital, The First Affiliated Hospital of Shenzhen University, Shenzhen, Guangdong, China

**Keywords:** ACL reconstruction, ACL rupture, cartilage lesion, osteoarthritis, risk factor

## Abstract

Anterior cruciate ligament injury is the most common sports injury in orthopaedics, which can adversely affect knee joint function and exercise of patients. Using arthroscopy to reconstruct the anterior cruciate ligament has become the first choice for treating anterior cruciate ligament rupture. However, different degrees of articular cartilage injury of the knee can be observed in patients after anterior cruciate ligament reconstruction. More importantly, the articular cartilage injury after anterior cruciate ligament reconstruction indicates that it will develop into osteoarthritis in the long term. It is of great significance to fully understand the factors that lead to the occurrence and development of cartilage injury. This article reviews the effects of surgical methods, meniscus status, different grafts, time from injury to surgical intervention, postoperative knee joint stability, postoperative rehabilitation, knee joint anatomical factors, and demographic characteristics of patients on articular cartilage degeneration after anterior cruciate ligament reconstruction. The present review provides insights into the anterior cruciate ligament reconstruction, which can be used to investigate new treatment strategies to delay and prevent the progress of osteoarthritis. At the same time, it provides a holistic understanding of the influence of multiple factors on cartilage lesions after anterior cruciate ligament reconstruction.

## Introduction

Anterior cruciate ligament (ACL) is the main stabilizing structure of the knee joint, which can not only prevent the tibia from moving forward but also have the functions of distributing energy and load within the joint, preventing internal rotation of the tibia. ACL injury is a common problem, and if improperly treated, it will seriously affect knee motion function, induce early knee osteoarthritis (OA), decrease quality of life, and in severe cases lead to disability of the joint, thus placing a huge burden on patients, families, and society (Barenius et al., 2014). Approximately 30% of patients who have a torn ACL will develop OA within 10 years with or without ACL reconstruction (ACLR) (Luc et al., 2014). Furthermore, most patients will progress to OA within 15 years of injury (Segawa et al., 2001). Nevertheless, so far, there is no effective treatment that can delay the progress of OA. One of the crucial reasons is the lack of effective methods to diagnose and detect early OA. The current view is that articular cartilage cannot be regenerated after injury. We only can alleviate the progress of cartilage degeneration to a certain extent (Intema et al., 2010). Therefore, early diagnosis and identifying factors associated with degeneration of articular cartilage are effective ways to control disease progression and reduce its harmful effects. In this paper, we review the effects of the patient’s general condition, intervention time (from ACL injury to surgery), ACLR surgery modality (single bundle, double bundle, selected graft, surgical approach), the state of intra-articular structures at the time of surgery, anatomical factors of the knee joint, postoperative knee stability, and postoperative rehabilitation to provide a theoretical basis for the prevention of cartilage damage after reconstructive surgery.

## Risk factors of cartilage lesion

### Patient’s general condition

The population of ACLR patients is widely distributed, and its demographic characteristics also influence changes in knee cartilage; for example, a high body mass index (BMI) is considered a risk factor for patellofemoral and tibiofemoral OA (Culvenor et al., 2015). Patterson et al. (2018) found a 2-fold increase in the incidence of OA in the knee joint at BMI >25. Another study showed that higher BMI was associated with medial compartment knee OA (Jones et al., 2019), and obesity might be associated with OA at the biomechanical and biochemical levels (Rai & Sandell, 2011; Wluka et al., 2013). Thus, for patients who prepare for surgery, maintaining a normal weight is essential for the knee’s cartilage. In addition, Newman et al. (2015) showed that the risk of secondary cartilage injury is higher in patients aged 14–19 years compared to patients aged <14 years. A study by Hiranaka et al. (2020) also found that being female gender and being aged >30 years relevant factors for cartilage degeneration. However, another study found that gender and age were not associated with cartilage damage (Shino et al., 1993); hence, there is no conclusive evidence regarding the effect of age and gender.

Meanwhile, we must consider the impact of other professions, such as the military community (Sun et al., 2022). The frequency of ACL injuries was ten times higher in the military than in the general population (Owens et al., 2007), and OA rates were much higher (Cameron et al., 2011). The general population’s risk factors for cartilage degradation do not entirely apply to this particular demographic.

### Intervention time from anterior cruciate ligament injury to surgery

ACL rupture in children and adolescents was previously considered a rare condition. However, as more and more children and adolescents participate in competitive sports, the incidence of ACL rupture is increasing. Clinicians are often faced with the dilemma of choosing treatment for ACL ruptures in skeletally immature patients. Early ligament reconstruction may result in angulation and shortening of the limb, while conservative treatment or surgery after epiphyseal closure may result in continued instability of the knee joint and secondary meniscal or articular cartilage damage. Several studies have concluded that early ligament reconstruction for ACL injuries in adolescents is associated with a lower incidence of secondary meniscal and articular cartilage injury, while delayed reconstruction is associated with a higher incidence (almost twice as high than in the early surgery group) (Lawrence et al., 2011; Anderson & Anderson, 2015). This may be due to repetitive micromotion during exercise with an unstable knee (Fehnel & Johnson, 2000). Therefore, early surgical treatment should be performed in children and adolescents with confirmed ACL.

Prodromidis et al. (2021) evaluated the incidence and grading of cartilage injury in adult patients with ACL injuries who underwent ACLR at 3, 6, 12, and >12 months. They showed that delayed ACLR was associated with an increased incidence of cartilage injury or degeneration. Thus, ACLR should be performed as early as possible after ACL injury, preferably within 3 months or after the inflammatory response has subsided. Furthermore, Taketomi et al. (2018) concluded that ACLR should be performed within 6 months after the injury to prevent cartilage degeneration. In general, ACL tear is an important trigger for the early onset of OA of the knee. Early surgery can prevent the development of concurrent intra-articular injuries (Chalmers et al., 2014) and delay the onset of knee OA (Richmond et al., 2011); therefore, aggressive treatment is recommended for patients after injury.

### The influence of surgical methods

The purpose of ACLR is to restore knee joint function and prevent joint instability, and repeated injuries to articular cartilage and other soft tissues. However, no research has shown that ACLR can prevent the occurrence of posttraumatic OA. A study showed that 17–20 years after injury, patients with and without ACLR showed joint degeneration on X-ray films (Koster et al., 2018). Half of the patients who received surgery had mild joint degeneration, while 16.5% had severe OA. However, 56% of untreated patients suffered from severe OA. We believe that ACLR does not fully prevent OA but at least reduces its incidence. Hiranaka et al. (2019) demonstrated that the second look at patients after ACLR showed that the articular cartilage of all compartments degenerated except for the lateral tibial plateau. Although the stability of the knee joint and clinical cure after reconstruction were desirable, OA in the patellofemoral and compartments begins to progress early after the operation. In addition, the choice of reconstructive procedure is also a factor affecting the progression of articular cartilage degeneration. Wang et al. (2011) found that double-bundle ACLR could reduce the injury of trochlear femoral cartilage in the short term after the operation compared with single-bundle ACLR, and the same conclusion was reached by Gong et al. (2013). Double-bundle reconstruction has been shown to result in better anterior-posterior and rotational stability (Jiang et al., 2012). Tajima et al. (2010) demonstrated through cadaveric studies that double-bundle reconstruction could better restore the normal contact area and pressure of the patellofemoral joint, thus reducing cartilage damage. As a traditional technique, single-bundle ACLR through the trans-tibial technique often leads to tunnel dislocation (Kopf et al., 2010). The placement of this non-anatomical tunnel may lead to abnormal knee joint kinematics after ACLR (Tashman et al., 2004), which may be a potential cause of cartilage degeneration (Van Eck & Fu, 2011). Andrä et al. (2021), however, found that tibial tunnel localization had a greater effect on cartilage degeneration progression than femoral tunnel localization. Patients who underwent revision ACLR exhibited a higher risk of progression of OA compared to those who underwent initial reconstruction (Grassi et al., 2016). Mitchell et al. (2018) found that the incidence of femoral condyle cartilage defects was higher in patients who underwent revision ACLR. Yoon et al. (2020) also found that as the frequency of ACLR injuries increased in individual patients, the incidence or severity of cartilage injury increased, as would the likelihood of repeated trauma or more prolonged persistent instability.

When the ACL is ruptured, it is usually accompanied by cartilage injury. It is necessary to deal with the injured cartilage during the reconstruction. Røtterud et al. (2016) followed up ACLR patients with full-thickness cartilage injury for 2 years and found that microfracture (MF) treatment had a negative effect on the knee osteoarthritis outcome score (KOOS), while debridement showed neither positive nor negative significant effects. The authors point out that this result may be due to biomechanical or biochemical abnormalities after ACLR, which could be unfavorable for MF, while joint debridement relieves symptoms by removing unstable cartilage. Hence, MF treatment is not well suited for ACL-reconstructed knees.

By pretensioning and preconditioning, Marchiori et al. (2021) discovered that different graft kinds and diameters displayed varying stress-relaxation, which is a mechanical characteristic linked to knee laxity. In order to lessen knee joint instability after ACLR, the current literature reveals the potential positive effects of pretensioning and preconditioning, including lowering graft elongation and maintaining graft tension and stiffness after graft fixation (Jisa et al., 2016). According to our speculation, improper pretensioning and preconditioning may also be contributing causes to cartilage deterioration. Future research should focus on biomechanical and histological studies, multidisciplinary collaboration, and clinical follow-up to discover the most effective pretensioning and preconditioning techniques.

### Graft selection

The best type of graft used in ACLR has been controversial (Biau et al., 2006). Each available graft option for ACLR has its advantages and disadvantages. Therefore, it is essential to choose the graft carefully for each patient. In the international ACL Study Group, most surgeons preferred autograft (Arnold et al., 2021). In 1992, the autogenous bone–patellar tendon–bone (BTB) graft was the most common choice for treating primary ACLR (nearly 90% of cases). Later, autogenous hamstring tendon (HT) grafts became increasingly popular, gradually 50% (Arnold et al., 2021). Since 2014, autogenous quadriceps tendon grafts have become more widespread (Arnold et al., 2021). Pinczewski et al. (2007) found that the incidence of OA after ACLR with patellar ligaments is higher than that after ACLR with HT. Sajovic et al. (2011) came to the same conclusion, which is not surprising because the graft comes from the patellar tendon, which affects the patellofemoral joint. However, Webster et al. (2016) performed a 15-year follow-up study and found that ACLR using BTB or HT grafts was associated with a comparable degree of cartilage degeneration and progression of OA. The same results were found in a study by another group (Holm et al., 2010). Interestingly, it has been shown that when combined with a medial meniscus injury, ACLR with an HT graft is more likely to cause cartilage degeneration and ultimately OA than ACLR with a BTB graft (Cantin et al., 2016).

Compared with autologous tendons, allogeneic tendons have the advantages of a shorter surgical time, small incision, and a wide range of tendon sources, which compensate for the limitations of autologous tendons. However, a study by Magnussen et al. (2018) found that ACLR using allografts was 15 times more likely to lead to patellofemoral cartilage injury than ACLR using autologous patellar tendon grafts, which is due to the delayed remodelling time of allograft. This leads to a decrease in long-term stability and mechanical function, which eventually leads to an increase in articular cartilage load and cartilage injury (Scheffler et al., 2008). For the athletes who want to return to the stadium and elderly patients who want to restore their exercise ability as soon as possible, the ligament advanced reinforcement system (LARS) has become the best choice. With the extensive use of LARS in ACLR treatment, more attention has been paid to its effect on articular cartilage. Tiefenboeck et al. (2015) found that 7 of 11 young patients showed radiological signs of OA osteoarthritis during a follow-up of at least 10 years after ACLR with LARS.

### The state of intra-articular structures at the time of surgery

The causes of knee cartilage injury in patients with ACL injury combined with meniscal injury are quite complicated. The injured meniscus causes shear and wear of the knee cartilage, which changes the pressure distribution of cartilage and causes cartilage injury. Dumont et al. (2012) found that patients with meniscal tears had a higher risk of cartilage injury. In addition, a meta-analysis of 16 studies with at least 10 years of follow-up confirmed that meniscectomy increased the risk of cartilage injury and OA after ACLR (Claes et al., 2013). It was found that partial meniscectomy was associated with an increased incidence of new cartilage defects compared with the meniscus repair and intact meniscus groups; patients with meniscal repair and intact meniscus had 64%–84% less chance of cartilage damage, respectively (Brophy et al., 2015). Using second look arthroscopy, Nakamae et al. (2018) further confirmed that partial meniscectomy was closely linked to the development of articular cartilage injury. Logan et al. (2019) found from a cadaveric study that resection of medial meniscus bucket-handle tear during ACLR would result in a significant increase in average and peak contact pressure of the medial and lateral menisci. By suturing the bucket handle, the biomechanics and kinematics of the knee joint can be restored more closely to the natural state, which would significantly reduce the incidence of cartilage degeneration and OA (Logan et al., 2019).

In addition, the location of the meniscal injury also directly affects the articular cartilage. When the meniscus injury occurs in the posterolateral horn, the cartilage of the lateral tibial plateau is damaged regardless of whether the meniscus is healed (Tsujii et al., 2019). Michalitsis et al. (2017) found a correlation between medial meniscus surgery and new cartilage defects in the medial and lateral femoral condyles, with the most severe cartilage damage in the lateral femoral condyle. Other studies have shown that patients with ACL rupture combined with popliteomeniscal fascicle tear showed more cartilage degeneration in the lateral compartment than those with ACL rupture alone 2 years after ACLR (Guimaraes et al., 2018).

Interestingly, it has been suggested that there is no correlation between meniscal injury and degenerative cartilage changes in some studies, which have shown that patients with ACL combined with meniscal injury only had cartilage remodelling rather than further degeneration in joint surfaces in the short-term postoperative follow-up. However, it remains uncertain whether this change is the beginning of joint degeneration (Asano et al., 2004). We believe that the reason for this discrepancy lies in the study’s heterogeneity of different surgical techniques, follow-up times and functional scores. Nevertheless, most scholars agree that there is a correlation between meniscal injury and cartilage degeneration. Therefore, preserving the integrity of the meniscus remains an important aspect of ACLR.

### Anatomical factors of the knee joint

Anatomical factors of the knee affect the changes in knee cartilage after ACL surgery. The more the inversion of the knee, the higher the incidence of cartilage injury in the medial compartment. However, the incidence of cartilage injury in the lateral compartment is independent of the lower extremity force line (Brophy et al., 2015). In acute ACL injuries, the incidence of severe cartilage damage ranges from 16% to 46% (Brophy et al., 2010); furthermore, studies have shown that the moment of ACL injury is the moment when the process of knee cartilage degeneration begins (Kia et al., 2020).

Chen et al. (2018) found that the contact area on the medial surface of the tibia was significantly smaller and more posteriorly positioned during knee flexion and extension activities on the injured side of ACL compared to the contralateral side. Murrell et al. (2001) found the highest rate of cartilage damage in the medial femoral condyle and the medial tibial plateau after ACL injury, and they attributed this to the abnormal mechanical loading present, leading to alterations in the cartilage structure of the tibiofemoral and patellofemoral joints.

The longitudinal assessment of patellofemoral alignment and morphology after ACLR by Macri et al. (2019) revealed that abnormal patellofemoral alignment was widespread 1 year after ACLR, with bisect offset pairwise fractional deviations of up to 14% and lateral patellar tilt of 53%, while morphological abnormalities were less common. The lateral displacement of the patella increased trochlear angle, and the shallower trochlear sulcus angle after ACLR was found to be associated with an increased risk of patellofemoral cartilage degeneration at 4 years (Macri et al., 2018). Liao et al. (2021) concluded that excessive lateral deviation of the patella after ACLR reduces the patellofemoral joint contact area, which further increases patellofemoral joint stress.

Moreover, the authors concluded that initial patellar malalignment, although in the short-term, can make the joint more susceptible to an adverse environment of elevated pressure and eventually induce patellofemoral joint OA (Liao et al., 2020). In fact, after ACL injury, the patella is rotated and tilted abnormally, which directly leads to a lateral shift of the patellofemoral cartilage contact area, which is a condition that cannot be restored to its initial state even by ACLR (Van de Velde et al., 2008). Compared to the tibiofemoral joint, there is evidence of a high percentage of patellofemoral degeneration after ACLR (Wang et al., 2011; Culvenor et al., 2013). Gong et al. (2013), Wang et al. (2015) found that the patellofemoral articular cartilage was the most severely damaged region. These biomechanical changes in the patellofemoral joint may predict the degeneration of articular cartilage and the development of OA.

In addition, Hart et al. (2022) found that the presence of a larger subpatellar fat pad and Hoffa’s synovitis after ACLR would increase the patient’s odds of having patellofemoral and tibiofemoral cartilage injury at 1 year post-operatively. At 5 years post-operatively, this chance was even seven times as high. In addition, studies have shown that the presence of effusion 1 year after ACL injury may be an indirect effect of OA. This inflammatory process may play a role in initiating degenerative changes (van Meer et al., 2016).

Furthermore, the degeneration of the femoral and tibial cartilage in the lateral compartment is related to the angle formed by the Blumensaat line and the posterior cortical extension line of the femur, the standard value of which lies between 23° and 60°. As the angle decreases, there is an increased risk of graft impingement, which in turn increases tibiofemoral and patellofemoral contact load and ultimately accelerates cartilage degeneration (Shelbourne et al., 2017). Mitchell found that the decrease of tibial plateau posterior inclination was related to medial femoral compartment cartilage injury (Mitchell et al., 2018), which was different from previous studies, which showed increased tibial plateau posterior inclination caused cartilage injury (Khan et al., 2014). However, the mechanism leading to this difference has not been clarified.

### Postoperative knee stability

Anterior–posterior stability and rotational stability of the knee joint after ACLR alone are not as good as before. Studies have shown that those with a sustained positive pivot shift test are more likely to develop knee OA postoperatively (Jonsson et al., 2004; Curado et al., 2020). Zampeli et al. (2021) found that sustained tibial rotation abnormalities were significantly associated with the development of articular cartilage degeneration by following patients after ACLR for an average period of 8.4 years. Cartilage degeneration was mainly located in the central region of the lateral femoral condyle, followed by the central and anterior regions, and most cases are superficial cartilage damage. These findings suggest that abnormal rotational motion is a potential risk factor for the development of postoperative traumatic articular cartilage degeneration after ACLR.

However, Asano et al. (2004) assessed the anterior–posterior stability and residual laxity of the knee after surgery, and found no correlation between the bilateral differences of the KT-1000 test (less than 0 mm, 0–3 mm, and>3 mm) and the progression of cartilage degeneration in the knee in the short term after ACLR. Several studies have also reported that residual anterior laxity does not affect the progression of degenerative cartilage changes (Küllmer et al., 1994). In contrast, some other authors reported the opposite conclusion (Struewer et al., 2012; Krutsch et al., 2017).

### Postoperative rehabilitation

Currently, rehabilitation protocols are more aggressive than in the past, requiring passive achievement of full-range knee angulation, immediate participation in activities, immediate partial weight bearing, and functional training (Adams et al., 2012). However, defining “return to sports” solely in terms of time factors does not guarantee optimal knee performance. Patterson et al. followed the sports performance of 78 patients 1 year after ACLR. They found that only one-fifth of patients met the criteria for generalized functional performance (limb symmetry index >90%), and patients with inadequate sports performance were at increased risk of patellofemoral bone contusion in a repeated test of four triple jumps (Patterson et al., 2020). Culvenor et al. (2018) analyzed the accelerated return to sports group (return to sports after <10 months vs. return to sports after ≥10 months or no return to sport) in the entire cohort and showed that early return to sport after ACLR, particularly in patients with inadequate extremity function, was associated with progression of early knee OA. Wang et al. (2015) performed standardized bilateral quadriceps isometric muscle strength tests in patients during follow-up after double-bundle ACLR (mean follow-up period, 24.1 months; range, 12–51 months). The results showed that significant degeneration of both patellar and trochlear cartilage was seen in the group with ≥20% decrease in peak quadriceps muscle torque compared to the healthy knee group, and only trochlear cartilage degeneration was seen in the group with <20% decrease in peak quadriceps muscle torque, while the incidence of patellar cartilage degeneration was significantly lower. The authors concluded that in the short term after ACLR, quadriceps strength recovery of more than 80% has a protective effect on patellar cartilage and is associated with less serious damage to the patellar cartilage (Wang et al., 2015). Friedman et al. (2021) discovered that increased activity 3 years after ACLR, particularly returning to Marx activity levels of greater than eleven, was substantially associated with an increased incidence of medial compartment posttraumatic osteoarthritis. Athletes with at least one weekly exercise requiring some combination of running, cutting, decelerating, and pivoting have Marx scores above eleven.

In summary, for some patients with poor postoperative knee function, return to exercise should be delayed appropriately and reduce high-intensity exercise to avoid further damage to the knee cartilage.

## Future outlook

Further research on the causes of cartilage damage following ACLR is still necessary. 1) This review thoroughly outlines the important causes of cartilage lesions, taking into account both well-known causes and some less-common ones (e.g., anatomical factors of the knee joint, postoperative rehabilitation). A thorough understanding of the risk factors impacting cartilage lesions is handy for readers. 2) To assist researchers in determining where to focus their future study, the influencing elements (including risk factors and protective factors) mentioned in the literature are directly listed and categorized in this review. 3) The review’s Table 1 lists the many study topics and key points in chronological sequence, which makes it easier for us to understand the overall direction of the research. At the same time, the number of citations in the article is added to facilitate readers to identify contradictory views in different literature.

**TABLE 1:** Risk factors of Cartilage lesion after anterior cruciate ligament reconstruction.

Factor	Author	Year	Citation	Number	Follow-up period	Commentaries
Demographic	Patterson et al. (2018)	2018	33	78	5 years	High BMI (>25 kg/m^2^) (+)
	Culvenor et al. (2015)	2015	140	111	1 year	BMI >25 kg/m^2^ (+)
	Jones et al. (2019)	2019	14	421	2 years	Higher BMI (+)
	van Meer et al. (2016)	2016	55	143	2 years	male (+)
	Hiranaka et al. (2020)	2020	6	40	16 months	Female (+)
	Asano et al. (2004)	2004	109	105	15 months	Patient’s age (+)
	Newman et al. (2015)	2015	79	231	NS	Age 14–19 years (versus <14 years) (+)
	Jones et al. (2019)	2019	14	421	2 years	Older age (+)
	Hiranaka et al. (2020)	2020	6	40	16 months	Age >30 years (+)
Intervention time	Murrell et al. (2001)	2001	156	130	NS	Waited a long time to have ACL reconstruction (+)
	Lawrence et al. (2011)	2011	94	70	NS	Delay in treatment of over 12 weeks (+)
	Anderson & Anderson, (2015)	2015	216	62	NS	Delayed ACLR (+)
	Shelbourne et al. (2017)	2017	56	423	22.5 ± 2.1 years	Older age at surgery (+)
	Taketomi et al. (2018)	2018	14	226	NS	Reconstruction performed within approximately 6 months (−)
	Curado et al. (2020)	2020	16	182	22 years	Age >30 years at surgery (+)
	Prodromidis et al. (2021)	2021	4	3559	NS	Surgery ≤3 months after injury (−)
Surgical methods	Shino et al. (1993)	1993	142	187	3–80 months	Conventional medial parapatellar incision (−)
						Use of the central one-third of the autogenous patellar tendon graft (+)
	Van Eck & Fu, (2011)	2011	7	NS	NS	non-anatomical tunnel (+)
	Tajima et al. (2010)	2010	41	7 cadaveric knees	NS	Anatomic DB ACLR (−)
	Wang et al. (2011)	2011	-	99	14 months	DB ACLR decrease the trochlea cartilage degeneration
	Gong et al. (2013)	2013	26	52	17.3–18.2 months	DB ALCR led to less cartilage damage
	Grassi et al. (2016)	2016	56	713	NS	Revision ACLR (+)
	Mitchell et al. (2018)	2018	33	487	NS	Revision ACLR (+)
	Yoon et al. (2020)	2020	10	82	minimum 2-year	Re-revision ACLR (+)
The state of the meniscus/cartilage	Dumont et al. (2012)	2012	171	370	NS	Medial and lateral meniscal tears (+)
	Claes et al. (2013)	2013	178	1554	NS	Meniscal resection (+)
	Culvenor et al. (2015)	2015	140	111	1 year	Meniscectomy (+)
	van Meer et al. (2016)	2016	55	143	2 years	Concomitant medial cartilage defect and meniscal injury (+)
	Shelbourne et al. (2017)	2017	56	423	22.5 ± 2.1 years	Medial meniscectomy (+)
	Michalitsis et al. (2017)	2017	10	29	2 years	Partial meniscectomy (+)
	Nakamae et al. (2018)	2018	13	29	2 years	Partial meniscectomy (+)
	Guimaraes et al. (2018)	2018	9	57	2 years	With popliteomeniscal fascicle lesions (+)
	Logan et al. (2019)	2019	12	10 cadaveric knees	NS	Excision of a bucket-handle medial meniscus tear (+)
	Curado et al. (2020)	2020	16	182	22 years	Medial or lateral meniscectomy (+)
	van Meer et al. (2016)	2016	55	143	2 years	persistent bone marrow lesions in the medial tibiofemoral compartment (+)
	Kia et al. (2020)	2020	8	40	NS	Size of initial bone bruise (+)
	Hart et al. (2022)	2022	3	131	Over 5 years	Hoffa-synovitis (+)
Anatomical factors	Khan et al. (2014)	2014	7	93	NS	Increased lateral tibial slope (+)
	Mitchell et al. (2018)	2018	33	487	NS	Decreased tibial slope (+)
	van Meer et al. (2016)	2016	55	143	2 years	joint effusion (+)
	Shelbourne et al. (2017)	2017	56	423	22.5 ± 2.1 years	Knee extension loss (+)
	Macri et al. (2018)	2018	18	111	1 year	Patellofemoral malalignment (+)
						altered trochlear morphology (+)
	Macri et al. (2019)	2019	13	73	5 years	Patellar lateral displacement (+)
						Patellar lateral tilt (+)
						Patellar morphology (+)
	Hart et al. (2022)	2022	3	131	Over 5 years	Infrapatellar fat pad volume (+)
Stability	Jonsson et al. (2004)	2004	269	63	5–9 years	Positive pivot shift (+)
	Struewer et al. (2012)	2012	90	73	13.5 years	Increased anterior laxity at long-term follow-up (+)
	Curado et al. (2020)	2020	16	182	22 years	Residual laxity (+)
	Zampeli et al. (2021)	2021	2	17	6 years	Abnormally increased tibial rotation (+)
Rehabilitation procedure	Wang et al. (2015)	2015	23	88	24 months	Greater than 80% recovery of quadriceps strength (-)
	Culvenor et al. (2018)	2018	16	111	1 year	Early return to sport (+)
						With poor lower limb function (+)
	Curado et al. (2020)	2020	16	182	22 years	Engaging in a pivoting sport (+)
	Patterson et al. (2020)	2020	13	78	1 year	A triple-crossover hop <90% LSI (+)
						Poor functional performance on the battery (all four tests <90% LSI) after 1 year post-ACLR (+)

(+) risk factor; (−) protect factor; NS, not shown; DB, double bundle; ACLR, anterior cruciation ligament reconstruction; BMI, body mass index; LSI, limb symmetry index.

## Conclusion

Despite the fact that ACLR cannot stop cartilage deterioration, it is still one of the best therapies available. In the past, we focused more on enhancing the stability of the knee joint following surgery to reduce articular cartilage deterioration. Previous research, however, has shown that restoring the stability of the knee joint is insufficient, and we must focus more on the specific risk factors causing cartilage deterioration. Early management is necessary to prevent accelerated cartilage deterioration when many high-risk variables are present.

## Abbreviations

ACL, Anterior cruciate ligaments; OA, osteoarthritis; ACLR, anterior cruciate ligaments reconstruction; BMI, body mass index; MF, microfracture; KOOS, knee osteoarthritis outcome score; BTB, bone-patellar tendon-bone; HT, hamstring tendon; QT, quadriceps tendon; LARS, ligament advanced reinforcement system.
